# Autism and detecting verbal truth and lies: veracity decisions, cue selection, judgment confidence, and self-report reasoning

**DOI:** 10.3389/fpsyg.2026.1871792

**Published:** 2026-08-26

**Authors:** Coral J. Dando, Tom Buchanan, Katie Maras

**Affiliations:** 1 Department of Psychology, School of Social Sciences, University of Westminster, London, United Kingdom; 2 Centre for Applied Autism Research, The University of Bath, Bath, United Kingdom

**Keywords:** autism, confidence, detecting deception, reasoning, truth and lies, veracity cue selection

## Abstract

**Introduction:**

Assessing veracity is a consequential task across many real-world settings, since judgments about truthfulness can shape important decisions. The way in which autistic individuals experience the social world potentially shapes how they make veracity decisions. Empirical research examining veracity judgments in autistic samples is sparse highlighting a clear gap in understanding how autistic observers make real-time veracity judgments following single exposure to an unfamiliar individual.

**Methods:**

We investigated the veracity decisions of 201 autistic and 193 not autistic adults from the general population, the cues they reported attending to, and using content analysis we explored reported reasoning. To approximate one feature of some real-world judgments, participants made a real-time veracity judgment following exposure to only one video clip of an interview with a mock suspect who was being either deceptive or truthful without feedback or opportunities for comparison.

**Analysis:**

Autistic observers were 1.47 times more likely to make a truth decision than non-autistic observers and overall were 1.51 times more likely to make an erroneous veracity decision. Both groups were comparably accurate when evaluating truth-tellers. Autistic participants noted reduced detail in deceptive accounts, and despite indicating the importance of verbal behaviours, most self-reported relying more on physical behaviours to guide their decisions. Autistic participants reported greater confidence overall in their veracity judgments.

**Discussion:**

Perceived discrepancies between the interviewee’s verbal and non-verbal communication were reported by many autistic participants. This may help explain an increased tendency toward truth judgments in this case, possibly resolving uncertainty by defaulting to honesty unless sufficient cues prompted a shift. Explicit, content-focused guidance regarding the diagnostic value of verbal behaviours for guiding veracity judgments may be beneficial.

## Introduction

Assessing veracity is a consequential task across many real-world settings, where judgments about truthfulness can shape important decisions and outcomes. Yet people are typically poor at detecting deception, a deliberate social communication behaviour where senders (deceivers) withhold and/or fabricate information to create in another (a receiver) a false belief (Bond and DePaulo, 2006; Dando et al., 2023; Dando and Ormerod, 2020; Vrij, 2019, 2022; Vrij et al., 2023). The psychological deception literature is extensive and spans multiple paradigms, but the conclusions are clear and typically consistent. Detecting truth and lies is inherently difficult and error-prone, with most individuals performing at around chance or only slightly above (approximately 54%) when distinguishing lies from truths (Bond and DePaulo, 2006; Levine, 2014, 2023; Markowitz, 2024). Furthermore, people tend to be overly confident, often believing they are ‘good’ at detecting deception even though confidence is not a reliable indicator of accuracy (e.g., Curci et al., 2018; Serra-Garcia and Gneezy, 2021; Schwardmann and Van der Weele, 2019). To date, however, relatively little is known about how autistic adults judge truth and lies, since current understanding is heavily centered on non-autistic populations. Autistic adult lay observers’ truth and lie judgments are the focus of the research reported here.

Autism is a lifelong neurodevelopmental condition characterized by differences in social communication and interaction, as well as focused interests and patterns of thinking that can shape how information is processed and interpreted (Lord et al., 2020). Estimated to occur in between 1 to 2% of the population (World Health Organization, 2022; Centre for Disease Control and Prevention, 2023), autistic adults’ understanding and experiences of the social world typically differ from those of non-autistic adults. Differences in social engagement during interactions, interpretation of behavioural cues and non-verbal gestures, and face processing can impact social contact and can sometimes trigger misunderstandings (Kurtz et al., 2025; Mosquera et al., 2021; McPartland et al., 2004; Nomi and Uddin, 2015; Reisinger et al., 2020). Given deception is a socially embedded act, these differences may shape how behaviours are perceived and evaluated by autistic observers when making veracity decisions.

### Detecting truth and lies

Detecting truth and lies is often difficult because individual, situational, and cultural differences mean liars do not display strong nor reliable non-verbal and/or verbal ‘signals’ to differentiate themselves from truth-tellers (e.g., Vrij et al., 2017, 2019). Observers consistently believe they can detect signs of deceit, typically relying on stereotypical pseudodiagnostic behaviours widely believed to be associated with truths and/or lies such as gaze aversion and self-adapting (e.g., Dando et al., 2024; DePaulo et al., 2003; Bond and DePaulo, 2006; Hartwig and Bond, 2011; Street and Masip, 2015; Vrij and Fisher, 2020; Vrij et al., 2019). More diagnostic indicators such as inconsistencies in verbal accounts or implausible levels of detail are often overlooked unless individuals are explicitly directed to attend to them. Liars frequently produce simplified or impoverished narratives that lack sensory, contextual, or plausible detail (Dando et al., 2024; Deeb, 2021; Vrij, 2022, 2023). In addition to a propensity to focus on pseudodiagnostic cues, there is a general cognitive tendency to presume that others are honest, which produces a systematic inclination to judge senders as truthful irrespective of behaviour (Levine, 2014; Pfänder and Altay, 2025; Reinhardt and Schindler, 2025). While this truth bias may be adaptive in everyday social interactions, it reduces veracity judgment accuracy, particularly when senders are motivated to mislead.

The cues that autistic adults attend to cues when judging truth and lies is underexplored. Differences in social communication and information processing styles may confer advantages, since autistic individuals may be less distracted by stereotypical pseudodiagnostic social behaviours. Certain autistic characteristics, including a tendency to have a more systematic approach to information gathering and a preference for analytical reasoning (e.g., Brosnan and Ashwin, 2023a,b) which may influence how available cues are weighted during veracity judgments, although some recent evidence suggests these differences may be context dependent rather than reflecting globally enhanced deliberative reasoning (Bastan et al., 2024, 2025). Autistic individuals may be naturally drawn to content-rich objective indicators such as level of detail, response plausibility, and the inclusion of verifiable event information which are among the more diagnostic cues that when systematically when attended to can improve veracity judgments (e.g., Dando et al., 2023; Dando and Ormerod, 2020; Granhag and Vrij, 2017; Sandham et al., 2022; Vrij et al., 2018, 2022).

There are grounds to argue that autistic individuals could be either better or worse at detecting deception. Some research suggests that autistic individuals may show a greater tendency toward analytic or deliberative responding in certain judgment tasks (e.g., Morsanyi and Hamilton, 2023). This has been interpreted as reflecting differences in information-processing style, including a greater focus on rule-based reasoning or systematic evaluation in some contexts. Such differences may influence how behaviour is weighted when making veracity judgments, potentially reducing reliance on appearance-based impressions or expectation-driven assumptions, reflecting differences in interpreting social cues, rather than reduced sensitivity *per se* (Lim et al., 2022). Focusing on the ‘message’ rather than the behaviours may improve veracity judgments (Buchanan et al., 2025; Martlew et al., 2025; Sandham et al., 2022). However, autistic individuals can sometimes be more compliant than neurotypical counterparts (e.g., Chandler et al., 2019; North et al., 2008, but see Maras and Bowler, 2012) and so may be susceptible to manipulation and exploitation in some instances. Face-to-face interactions may place greater social demands on autistic receivers, making the detection of deception more challenging (Bagnall et al., 2022; Dando et al., 2023). Switching between alternative perspectives and understanding and predicting others’ mental states are believed essential for improved lie detection but can be difficult for autistic individuals (e.g., Livingston and Happé, 2017; Penuelas-Calvo et al., 2019).

Understanding autistic individuals’ veracity judgment behaviours is important, since in the UK (and elsewhere) adults with an autism diagnosis may regularly make such judgments. Furthermore, autistic individuals are employed in professional roles where assessing credibility is a day-to-day activity (e.g., as police officers). As far as we are aware there are just a handful of empirical truth and lie studies involving autistic adults (e.g., Brewer et al., 2023; Michael and Brewer, 2025; Williams et al., 2018; Kurtz et al., 2025; Van Tiel et al., 2021), but none mirror the situations in which some veracity judgments are made in daily life, where people make real time judgments about people whom they do not know in the absence of guidance/training. In such settings, receivers cannot make relative judgments (comparing behaviours across or within groups) and are unable to pose questions. For example, deciding whether to trust a social media post, or engage with members of a new group or organization following a recruitment campaign, judging the veracity of news reports, or make a financial investment following a sales ‘pitch’. Similarly in more formal lay-person contexts such as courtroom decision-making (juries), assessment panels (lay-members/lay-visitors), and in boardroom situations (non-executive directors).

Prior research examining deception-related judgments in autistic samples has yielded mixed findings. In repeated-trial video paradigms, autistic observers have shown reduced lie-detection accuracy relative to matched non-autistic participants, while displaying a similar truth bias (Williams et al., 2018). However, participants were able to make relative judgments across multiple clips, and response opportunities were sometimes limited to brief yes/no/do not know decisions, reducing access to richer verbal cues. Other findings suggest that self-reported autistic traits may be associated with greater resistance to misleading demeanor cues under certain conditions (Kurtz et al., 2025). Deception-game paradigms have also found lower initial detection performance among autistic participants, alongside evidence of learning across repeated trials (Van Tiel et al., 2021). Prior vignette-based studies suggest that Theory of Mind difficulties and verbal ability may be associated with poorer discrimination of suspicious behaviours, but they have allowed relative judgments, although no practice effects were reported, and seemingly neither studies examined real-time veracity judgments *per se* (Brewer et al., 2023; Michael and Brewer, 2025). Taken together, existing findings suggest performance varies across paradigms and available cues, yet there remains a clear gap in understanding how autistic adults make real-time veracity judgments following a single exposure to an unfamiliar individual without opportunities for comparison or learning.

### The current study

This pre-registered study investigates veracity decisions made by autistic adults, truth and lie cues attended to, decision confidence, and asks for subjective assessments of interviewee responses to questions. To approximate one feature of some real-world judgments (single exposure without feedback) observers had access to just one video clip of a formal information gathering forensic style interview where the interviewee was either being truthful or deceptive, as might be seen by a member of the public sitting on a jury, parole board or a tribunal, for example. The literature for guiding the development of hypotheses is sparse, thus we developed a series of research questions, as follows, (i) how accurate are autistic adult observers in making veracity judgments, (ii) how do Autistic adult observers make truth and lie decisions, (iii) how confident are autistic observers in their veracity decisions, and (iv) do autistic adults recognize information rich and detailed verbal responses.

## Methods and materials

A 2 (group: autistic; non-autistic) X 2 (condition: truth; Lie) X 2 (video A; B) between subject experimental design was employed with 394 adults from the UK and US general populations participating as observers. The mean age was 33.47 years (SD 11.35) ranging from 18 to 75 years with 247 males and 134 females. Thirteen participants preferred not to say. Participants were monolingual English speakers and were paid £4.00. Two hundred and one autistic adults participated. Autism status was self-reported as diagnosed during childhood and not independently verified, which may introduce heterogeneity. Prolific’s embedded pre-screeners were used to recruit 171 autistic adults (98 were born and living in the UK, 78 born and living in the USA). The remaining 30 were all born and living in the UK and recruited via word of mouth and snowball sampling following contact with autism community groups and charities.

One hundred and ninety-three non-autistic adults from the general population participated: 120 were members of the Prolific research panel, the remainder were recruited via word of mouth and snowball sampling (83 were born and living in the USA, the remainder were born and living in the UK). The study used a between-subjects design. All participants were randomly assigned to one veracity condition (truth or lie). Within each veracity condition, participants were further randomly allocated to one of two video variants (A or B) to control for stimulus-specific effects. In the autistic group, 100 participants were assigned to the lie condition and 101 to the truth condition, with individuals in each condition randomly viewing either Video A or Video B. In the non-autistic group, 99 participants were assigned to the lie condition and 94 to the truth condition, again with random allocation to Video A or B within each condition. Each participant viewed only one interview video. The mean age of autistic participants was 33.4 years (SD = 11.15), and the mean age of non-autistic participants was 33.6 years (SD = 11.58). *A priori* power analysis for independent groups odds ratio analysis indicated a minimum sample size of 340 participants to achieve power of at least 0.80, with an alpha level of 0.05, and medium effect size (Faul et al., 2007). SPSS data file can be accessed at OSF.

*Autism Spectrum Quotient (AQ-10)*. The AQ-10 questionnaire comprises 10 descriptive statements about preferences and habits (see Hoekstra et al., 2011; Lundin et al., 2019; Murray et al., 2017). AQ − 10 score is calculated by totalling responses. Bimodal scoring system was used (range 0–10).

*Adult Theory of Mind (A-ToM)*. A quick, forced-choice version of A-ToM was used for group characterisation purposes, only (materials available from Brewer et al., 2017, 2023). Participants selected one forced-choice option as quickly as possible. Answers were scored as either 0 (incorrect) or 1 (correct). Scores for both the social and physical subscales, each range from 0 to 6. Higher scores indicate greater theory of mind. A-ToM is included as a measure to support group characterisation, not as a predictor variable, providing a descriptive confirmation of differences in Theory of Mind performance across groups.

*Stimuli video clips*. Four information gathering interview clips were selected from a larger data set, two for the lie condition and two for the truth condition, referred to as LA (lie interview A), LB (lie interview B), TA (truth interview A) and TB (truth interview B). Interviewers employed an empirically validated questioning technique known to amplify verbal veracity signals (Dando and Ormerod, 2020; Dando and Bull, 2011; Ormerod and Dando, 2015; Sandham et al., 2022). Interviews were of a similar duration, and all involved one female interviewee and one female interviewer. Interviewees were aged between 29 and 39 years. Interview durations ranged from 3 min 19 s to 3 min 46 s (*M*_duration_ = 3 min 31 Seconds). The Framed Controlled Cognitive Engagement (F-CCE) protocol was used, comprising two discrete interview phases. The first phase is the free account where interviewees are asked to provide a detailed and full explanation of what happened and who was involved. The second probing question phase followed, comprising 5 focused questions concerning visual, auditory, emotional and blame information, with the final question focusing on withholding and errors (see Dando and Ormerod, 2020 for interview protocol). Interviews were coded for a series of verbal content and verbal response behaviours exhibited by interviewees.

1. Event information items (unique) provided (detail)
2. Interviewee talking time
3. Do not know/cannot remember/did not see responses to questions

These verbal responses were selected since they map onto some of the more robust verbal and interactional veracity signals (see Bogaard et al., 2024; Dando et al., 2023; Palena et al., 2021; Vrij, 2022). Associated physical behaviours (body language) were not controlled nor counted since individuals are known to behave variously when being deceptive and/or telling the truth.

### Participants and procedures

*Interviewee Participants*. Interviewees were paid £25 to participate and were further incentivised to be deceptive or to be truthful about the events they had experienced when answering questions, with an additional payment of £50 if they complied with instructions. The interview stimuli used comprised two interviews with truthtellers and two with deceivers. Each interviewee had been involved in a live event involving a verbally violent argument between two (actor) individuals which culminated in a laptop computer being smashed. The event was designed to mimic the experiences of some individuals who may witness an event and are later be interviewed as a witness/person of interest. Interviewee performance data and the paradigm have been reported in full (see Dando and Ormerod, 2020; Dando et al., 2024; Sandham et al., 2022), but for replication purposes and manuscript clarity a description of the paradigm and interview procedure is provided in Supplementary materials.

*Observer Participants*. All observers accessed the Qualtrics platform using a one-time anonymous link and completed a series of demographic questions, watched/listened to the video interview and completed several veracity questions (forced choice, scale and open-ended questions), questions about how they had made their veracity decision (what behaviours had been influential etc.) and were asked to indicate confidence in their decision. Finally, all participants completed the AQ-10, followed by the A-ToM.

### Manipulation checks

*Interviewee Verbal Behaviours*. Truth and lie conditions differed as follows. Truthful interviewees spoke for almost double the amount of time than deceptive interviewees and provided approaching twice the number of unique event information items in response to questions. Interview transcripts were coded for information, which was counted once only (i.e., repetitions were ignored). For example, if an interviewee stated, ‘*the student picked up the table’* the ‘*student’* ‘*picked up*’ and ‘*table*’ utterances were coded as three event information items. Interviews were coded independently by two researchers naïve to the experimental design and research questions (see Dando and Ormerod, 2020; Hauch et al., 2016; Memon et al., 2010). Coders underwent a 4-h training session and worked several examples. Interclass correlation coefficient (ICC) analysis testing for absolute agreement between coders for all four interview segments was conducted. Mean estimations with 95% CI indicate good inter-rater reliability, ICC = 0.899 (95% CI, 0.593; 0.975). Truth tellers did not offer any ‘do not know’ nor ‘did not see’ responses and only one ‘cannot remember’ response. Liars responded, ‘do not know’, ‘did not see’ and ‘cannot remember’ ranging between two and five times more often than truth tellers (see Table 1).

**Table 1:** Characteristics of the interview stimuli.

Veracity condition	Interview	Talking time	“Do not know” responses	“Cannot remember” responses	“Didn’t see” responses	Unique information items
Lie	A	67.45	4	3	5	12
	B	77.33	3	4	4	11
Truth	A	148.29	0	0	1	24
	B	139.51	0	0	1	27

Talking time refers to the duration of each interview. “Do not know,” “cannot remember,” and “did not see” responses represent uncertainty statements made by interviewees during questioning. Unique information items refer to the number of distinct event information items contained within each interview.

*Interview Videos*. There were no associations between video (A; B) and veracity decision (truth: lie) *χ* ^2^ (1) = 3.180, *p* = 0.075 and veracity decision accuracy, χ ^2^ (1) = 0.153, *p* = 0.696 in truth and lie conditions. There were no significant differences between video (A; B) for detailedness and confidence ratings, *F* (1, 355) = 1.813, *p* = 0.179, and *F* (1, 392) = 2.502, *p* = 0.115, respectively. Thus, results below reported collapse video A and B responses as a function of veracity condition.

*Autism Spectrum Quotient 10*. AQ10 scores differed significantly across the two observer groups, *F* (1, 392) = 1057.462, *p* < 0.001, η_p_^2^ 0.73. Autistic participants scored higher (*M* = 7.51, SD = 1.50) than non-autistic participants (*M* = 2.39, SD = 1.63).

*Adult Theory of Mind*. Mean social (*M*_autistic social_ = 2.05, SD = 1.27; *M*_non-autistic social_ = 4.47, SD = 1.07) and physical (*M*_autistic physical_ = 2.54, SD = 1.05; *M*_non-autistic physical_ = 4.43, SD = 1.13) sub scale scores as a function of observer groups differed significantly, *t* (392) = −20.401, *p* < 0.001, and *t* (392) = −17.277, *p* < 0.001. Autistic participants scored lower on both scales that non-autistic participants.

## Results

### Analysis approach

Analysis was guided by research questions. Veracity judgments and judgment accuracy categorical data were analysed using loglinear analysis to examine frequency and relationships across autistic and non-autistic observers (research questions i and iv). Observer confidence (research question iii) and detailedness ratings (research question ii) both on a one to ten scale, and veracity cue selection on a one to six scale (research question ii) were analysed using a series of ANOVAs. Qualitative content analysis allowed a within-group exploration of autistic participants’ reported reasoning processes, in line with primary research question (ii) allowing us to interpret qualitative textual response data. We also report the non-autistic qualitative content analysis for descriptive and contextual purposes only. No between-group qualitative comparison was undertaken, as the principal focus of this paper is the autistic sample. Coding was conducted separately within each group, thus emergent codes were not identical, although some areas of overlap were observed (see the discussion).

### Veracity decision

Overall, a truth bias emerged with a similar truth bias pattern as a function of group and veracity condition (see Table 2). A three-way log-linear analysis examined veracity decision (truth, lie) as a function of group (autistic; non-autistic) x condition (truth; lie). K-way effects revealed the highest order group x condition x veracity decision interaction was significant, *χ* ^2^ (1) = 4.427, *p* = 0.036. Odds ratio indicated autistic participants in the lie condition were 2.28 times more likely to make a truth decision than non-autistic participants. All two-way associations between group (autistic; non-autistic) and veracity decision, and condition (truth/lie) and veracity decision, were non-significant, all *p*s > 0.075.

**Table 2:** Veracity judgments and decision accuracy as a function of veracity condition and participant group (*N* = 394).

Veracity condition	Participant group	Truth judgment	Lie judgment	Correct decisions	Incorrect decisions	N
Truth	Autistic	69 (68.3%)	32 (31.7%)	69 (68.3%)	32 (31.7%)	101
	Non-autistic	66 (70.2%)	28 (29.8%)	66 (70.2%)	28 (29.8%)	94
Lie	Autistic	74 (74.0%)	26 (26.0%)	26 (26.0%)	74 (74.0%)	100
	Non-autistic	55 (55.6%)	44 (44.4%)	45 (45.5%)	54 (54.5%)	99
Overall	Autistic	143 (71.1%)	58 (28.9%)	95 (47.3%)	106 (52.7%)	201
	Non-autistic	121 (62.7%)	72 (37.3%)	111 (57.5%)	82 (42.5%)	193
Total		264 (67.0%)	130 (33.0%)	206 (52.3%)	188 (47.7%)	394

Values are frequencies with row percentages in parentheses. Percentages are calculated within each participant group and veracity condition. Overall percentages are calculated within participant group.

### Veracity decision accuracy

Overall, veracity decision accuracy was close to chance level (50: 50) with 52.3% correct and 47.7% incorrect veracity decisions (see Table 2). A three-way loglinear analysis (group x veracity x accuracy) examined decision accuracy. The final model indicated a significant two-way higher order effect, χ ^2^ (3) = 50.613, *p* < 0.001. There was a significant association between group (autistic; non-autistic) and decision accuracy, χ ^2^ (1) = 5.181, *p* = 0.023. Odds ratio indicated that autistic participants were 1.51 times more likely to make an erroneous veracity decision. There was also a significant association between veracity condition (truth; lie) and decision accuracy, χ ^2^ (1) = 46.365, *p* < 0.001. All participants were 4.06 times more likely to make a correct veracity decision in the truth condition. All other associations were non-significant, all *p*s > 0.290.

### Confidence scale

Participants rated confidence in their decisions (1 to 10 scale). A 2 (group: autistic, non-autistic) x 2 (condition: truth; lie) x 2 (decision accuracy: correct; incorrect) ANOVA revealed a significant main effect of group (autistic: non-autistic), *F* (1, 386) = 52.951, *p* < 0.001, η_p_^2^ 0.12 (see Table 3). Autistic participants were more confident (*M* = 6.62, *SD* = 1.87, 95% CI, 6.47, 7.02) than non-autistic (*M* = 5.34, *SD* = 1.74, 95% CI, 5.06, 5.59). Main effects of veracity condition and accuracy of decisions, and all interactions were non-significant, all *F*s < 5.369 all *p*s > 0.021.

**Table 3:** Mean confidence and detailedness ratings as a function of participant group and veracity condition (*N* = 394).

Variable	Level	Detailedness M (SD), 95% CI	Confidence M (SD), 95% CI
Condition	Lie	4.52 (1.47), 4.31–4.73	6.20 (1.79), 5.94–6.44
	Truth	6.54 (1.58), 6.29–6.79	5.79 (2.02), 5.51–6.01
Participant group	Autistic	5.38 (1.88), 5.17–5.59	6.62 (1.87), 6.37–6.87
	Non-autistic	5.68 (1.72), 5.43–5.92	5.34 (1.74), 5.08–5.59
Participant group × condition	Autistic—lie	4.38 (1.46), 4.08–4.68	6.71 (1.75), 6.36–7.06
	Autistic—truth	6.38 (1.71), 6.08–6.67	6.53 (1.98), 6.18–6.89
	Non-autistic—lie	4.66 (1.47), 4.36–4.78	5.68 (1.68), 5.32–6.03
	Non-autistic—truth	6.70 (1.31), 6.31–6.77	4.99 (1.98), 4.62–5.35

### Detailedness scale

On a scale of 1 to 10 participants rated detailedness of accounts (see Table 3). A 2 (group: autistic, non-autistic) x 2 (condition: truth, lie) x 2 (decision accuracy: correct, incorrect) ANOVA revealed significant main effects of veracity condition, *F* (1, 349) = 92.998, *p* < 0.001, η_p_^2^ 0.22, and veracity decision accuracy, *F* (1, 349) = 18.776, *p* < 0.001, η_p_^2^ 0.05. Participants in the lie condition rated accounts as less detailed than those in the truth condition. Participants who made an accurate veracity decision rated accounts as more detailed (*M* = 5.89, *SD* = 1.89, 95% CI: 5.64, 6.10) than those who were inaccurate (*M* = 5.09, *SD* = 1.56, 95% CI: 4.83, 5.36). All other main effects and interactions were non-significant, all *p*s > 0.080 all *F*s < 3.084.

### Veracity cues

Participants indicated which cues they had attended to when making their veracity decision, choosing one response from a choice of six (1 = ONLY the way the person behaved; 2 = MAINLY how the person behaved, but I took some notice of was said; 3 = behaviour AND what was said were equally important; 4 = MAINLY what was said, but I took some notice of behaviour; 5 = ONLY what was said; 6 = I am unsure). Overall, the mean response when asked how participants had made their veracity decision was 3.42 (SD = 1.09) indicating that participants noted how people ‘*behaved AND what they said’* when making a veracity decision. A 2 (group: autistic, non-autistic) x 2 (condition: truth; lie) x 2 (decision accuracy: correct, incorrect) revealed a significant main effect of group, *F* (1, 386) = 71.013, *p* < 0.001, η_p_^2^ 0.16. Non-autistic participants indicated a tendency to note *‘mainly behaviour*, *only taking a little notice of what was said’* (*M* = 2.96, SD = 0.91). Autistic participants indicated that they tended to focus on *‘mainly what was said, only taking a little notice of behaviour’* (*M* = 3.89, SD = 1.08). Main effects of veracity condition and decision accuracy and all interactions were non-significant, all *p*s > 0.131 all *F*s < 2.283.

### Content analysis

Participants responded to two open questions (see Supplementary materials), henceforth Physical Cues (focused on physical behaviours) and Verbal Cues (focused on verbal behaviours). Conceptual content analysis (CCA) was employed to explore reported reasoning processes (e.g., Lyons and Coyle, 2021; Schreier, 2012). This approach was interpretative and exploratory rather than predictive or designed for formal between-group modelling. Nevertheless, it provided a useful means of identifying potential loci of group differences in veracity decisions. Of the 201 autistic and 193 non-autistic participants, 183 and 186, respectively, responded to both questions (Physical and Verbal).

An independent coder first identified explicit references to behaviours in responding to each of the questions (e.g., eye contact, hesitant voice, nervous). These micro-codes were then aggregated into a set of defined concept families (Lyons and Coyle, 2021; Schreier, 2012). A second coder independently coded all answers to each question, using the codebook. The codebook was agreed and refined via discussion and then applied to the full dataset. Codebook themes are described and illustrated by quotes, and the occurrence of each theme are counted (multiple mentions of the same behaviour were only counted once).

### Physical cues autistic respondents

Theme 1: *Personal beliefs and intuition* was the most common theme occurring in 70% of responses, referencing popular beliefs and classic ‘rules’ or a ‘personal framework’ linking a specific behaviour to veracity decisions. Here, heuristic beliefs regarding individual veracity indicators were applied automatically, assuming universal meaning.

Theme 2: *Narrative behaviour and speech patterns* was the second most common theme, occurring in 56% of responses. Despite the focus of this question being ‘*physical/movement behaviours’* many autistic respondents mentioned verbal behaviours, and so this theme encompasses observations related to auditory qualities of verbal responses, not *what* was said, but *how* it was said with a focus on how perceived quality and style of speech (tone, pauses, and mumbling) had guided decisions. For example, talking quickly was sometimes interpreted as nervousness, while speech that ‘flowed’ was viewed as more truthful. Filler words like “erm” and “uh” typically suggested deception.

Theme 3: *General disposition* was the least common theme, occurring in 39% of autistic responses centred on interviewee demeanour. General disposition was not judged in a binary way (honest vs. dishonest) but rather subjectively interpreted, suggesting veracity assessments may have been shaped by holistic impressions (Table 4).

**Table 4:** Autistic respondent’s physical cue themes 1, 2, and 3 exemplar quotes.

*Theme 1: Personal beliefs and intuition*
“Maintaining good eye contact signifies confidence and sincerity” (A-117).
“Avoiding eye contact might suggest discomfort or deceit” (A-107).
“Fidgeting or restlessness suggest nervousness or stress, linked to lying” (A-98).
“She crossed their legs but was relaxed, so truthful” (A-77).
“She was sitting on her hands. Defensive stance, so lying” (A-61).
“Rapid eye movement, so liar” (A-9).
“Body language was tense, arms folded, liar” (A-10)
*Theme 2: Narrative behaviour and speech patterns*
“Pauses. These are signs that the person is manipulating the truth.” (A-29)
“I could hear from her tone. I think the person is lying.” (A-154)
“Even though her body language was tense… I still think that what she was saying is exactly what happened, but the way she spoke was odd.” (A-60)
“…Tone of voice and manner of speaking just did not come off as genuine to me.” (A-19)
“Too many pauses for my liking, and when she did speak she just kept on going err, err. So she was a liar.” (A-79)
*Theme 3: General disposition*
“She appeared to be as calm as ever to me.” (A-39)
“She was acting very casual and conversational.” (A-22)
“I thought she seemed nervous but she seemed nice.” (A-90)
“The way she sat down and how she was composed while answering.” (A-71)
“She looked uncomfortable.” (A-32)
“She was very attentive and did not look dishonest.” (A-46)

### Physical cues not-autistic respondents

Theme 1: *Convergence of perceived credibility cues*. The most dominant theme (occurring in 78% of responses) illustrates how clusters of behaviours, (e.g., posture, facial expression, eye contact, gestures, and fidgeting) were used to assess credibility. Crossed arms, looking away, fidgeting, or touching the face were interpreted as indicators of deception, while relaxed posture, consistent eye contact, calm speech, and expressive gestures were typically associated with honesty.

Theme 2: *Narrative behaviour and speech patterns*. Despite the focus of this question being **‘***physical/movement behaviours,’* the second most common theme, occurring in 62% of responses to this question illustrates how detail and coherence in storytelling (verbal content) quality guided decisions, although physical behaviour was sometimes mentioned alongside verbal behaviours. Here, structure, coherence, and level of detail in the verbal account were viewed as truth indicators.

Theme 3: *Personal beliefs and intuition*. This theme occurred in just 29% of responses and captures how participants relied on their lived experiences, and intuitive impressions to make veracity judgments. Some defaulted to truth unless there was strong reason to suspect dishonesty (Table 5).

**Table 5:** Not autistic respondent’s physical cue themes 1, 2, and 3 exemplar quotes.

*Theme 1: Convergence of perceived credibility cues*
“She maintained steady eye contact… unhurried and calm demeanour and no gaze avoiding” (NA-89)
“Truth because they were very relaxed in the posture and did not look nervous or uncomfortable and so together this says truthful” (NA-141)
“Body language seemed very relaxed… unworried, calm and unhurried hand movements.” (NA- 101)
“Nervous smile when answering… Sitting on hands and her voice was lowered as if lacking confidence and scared” (NA-34)
“Laughing when telling about the incident, and touching her face frequently, fidgeting in the chair during the interview. Overall, this means liar” (NA-70)
*Theme 2: Narrative behaviour and speech patterns*
“The person interviewed looked defensive and gave clipped answers but opened up both in posture and in their more detailed account of what happened later in the interview. This made me want to believe them.” (NA-2)
“Mostly considered the speed in which she spoke… and how many unprompted details she provided.” (NA-28)
“She seemed open and gave very detailed information.” (NA-91)
“She also seemed quite rushed to get through her telling of what happened, like she wanted to move on from being questioned.” (NA-17) “Too slow. She was thinking far too much” (NA-141)
*Theme 3: Personal beliefs and intuition*
“I kind of always assume someone is telling the truth unless I have evidence that they aren’t.” (NA-76)
“She was telling the truth because she spoke how I speak when I am describing something that has happened.” (NA-16)
“I did not notice anything suspicious, but I had a gut feeling that she was lying.” (NA-106)
“What stuck out to me was the way she tried to appear too helpful—I do not know, something about it just felt off.” (NA-20)
“She reminded me of myself—awkward and soft-spoken. That made me feel she was probably just nervous, not lying.” (NA-43)
“I go with my first impression, and it is that she was truthful.” (NA-90)

### Verbal cues autistic respondents

Theme 1: *Conflict between words and body language/behaviour* was the most common theme occurring in 82% of responses, indicating perceived discrepancies between verbal and non-verbal behaviours. Perceived incongruence across communication channels (what is said vs. how it is said) resulted in veracity being questioned.

Theme 2: *Verbal inconsistency* occurred in 54% of responses indicating some autistic observers based their judgment on changes in the narrative and story content and how accounts evolved in response to questions. Consistency of the narrative seemed important since when details shifted between retellings, became increasingly exaggerated, or lacked clarity, these behaviours were interpreted as signs of fabrication (Table 6).

**Table 6:** Autistic respondent’s verbal cue themes 1 and 2 exemplar quotes.

*Theme 1: Conflict between words and body language/behaviour*
“I think the person was telling the truth, although her body language indicates otherwise.” (A-2). “The person started with an open body language… then it slowly went into a closed-off position and covering her mouth. But the story she told was very convincing so difficult for me” (A-8). “The story telling was quite fluid and consistent… However, the body language was tense, fidgety and closed off.” (A-102). “What she said was quite detailed and plausible and as the interview went on I felt that her account of what happened was truthful. But her body language was very fidgety and closed.” (A-81). “On one hand, her story was plausible. On the other hand, her body language was very closed off which is often interpreted as a sign of dishonesty.” (A-79). “Her words and body language were in conflict. She spoke confidently but behaved oddly” (A-101)
*Theme 2: Verbal inconsistency*
“The second time, her story was different, more extreme. This gave me the impression that she was lying.” (A-18).
“…The way that the story got slightly more odd each time.” (A-28).
“Some details were slightly different in the retelling…” (A-97).
“I feel the person was mostly honest but embellished the story a little bit…did not seem real.” (A-51).
“On balance I believed her - but with some reservation because the things she said later were often different.” (A-104).
“Inconsistencies in what she was saying when telling the story a few times…” (A-139).

### Verbal cues not autistic respondents

Theme 1: *Verbal inconsistency* occurred in 95% of non-autistic responses with a focus on changes in the account across repeated tellings. Differing accounts or responses to repeat questions were viewed as indicators of deception, suggesting consistency and story development over time were key veracity indicators for most respondents.

Theme 2: *Vague and effortful narrative* occurred in 72% of non-autistic responses. Pauses, vagueness, and effortful narration that did not flow were viewed as indicators of withholding, editing, or manipulating information and thus interpreted as evidence of deceit (Table 7).

**Table 7:** Not autistic respondent’s verbal cue themes 1 and 2 exemplar quotes.

*Theme 1: Verbal inconsistency*
“There were some minor inconsistencies in the retelling of the story, like extra details in the second account which could have been added to make the story sound more believable.” (NA-69) “She repeated phrases and mentioned that giving an account of the incident would involve repeating herself, which suggested that the story was tight and consistent.” (NA-141) “She repeated his account of what she heard twice when prompted. She seemed unhurried. This rings true to me.” (NA-190) “The pitch and story remain pretty consistent throughout the interview.” (NA-42) “Second time she told the story it was so very much different and the extreme lack of details gave her story away.” (NA-19)
*Theme 2: Vague and effortful narrative*
“She is definitely manipulating the truth and stopping herself telling the story. She was vague.” (NA-11)
“The first part of the interview had short, clipped responses which did not feel believable.” (NA-119) “She was very soft spoken and did not provide much detail…very vague.” (NA-77) “Holding words like um. And a load of them, so very vague information” (NA-157) ‘So much hesitation. Woolly and hard work. Liar!” (NA-17)

## Discussion

We were concerned with understanding veracity decisions made by autistic observers where the sender is unknown to the receiver and where the receiver cannot make comparative judgments across multiple trials, mimicking some day-to-day credibility judgment contexts. Overall, a truth bias emerged across our sample, with accuracy at around chance levels but with much improved accuracy in the truth-teller condition. These overall results mirror some of the truth and lie literature that suggests people tend to exhibit a truth bias, whereby they are more likely to judge statements as truthful, assuming honesty unless suspicion is actively triggered (Bond and DePaulo, 2006; Dando et al., 2023; Levine, 2014, 2023; Markowitz, 2024; Vrij et al., 2010; Vrij et al., 2019, 2022) with improved accuracy when judging truth-tellers (Bond and DePaulo, 2006; Hartwig and Bond, 2011; Street and Masip, 2015). However, several key findings and differences emerged when considering the two participant groups (autistic vs. non-autistic).

Irrespective of veracity condition, autistic observers were 1.47 times more likely to make a truth decision than non-autistic observers. Further, autistic observers and were 1.51 times more likely than non-autistic to make an erroneous veracity decision overall, although they were as accurate as non-autistic participants when judging truth-tellers. Autistic observers in the lie condition were 2.28 times more likely to default to the truth and thus make an incorrect decision than non-autistic participants and so may be more vulnerable to deception in some situations, although more research is needed as highlighted by the limitations of our study (see also Kurtz et al., 2025; Williams et al., 2018; Lim et al., 2022).

*Post hoc* qualitative self-reported reasoning data does not support direct comparisons and only provide perceptions of their reasoning, although there are some areas of overlap and conceptual convergence, the themes were qualitatively distinct across groups (Nowell et al., 2017). Nevertheless, these data offer some insights. Over 50% of autistic observers appeared sensitive to some of the more diagnostic verbal veracity cues. However, autistic participants frequently appeared conflicted, describing uncertainty when verbal content appeared incongruent with accompanying physical behaviour. This perceived across channel mismatch may have diluted the diagnostic value of potentially more valid verbal cues, particularly given that over 80% referred to perceived discrepancies between communication channels. Overall, autistic participants described attending to verbal behaviour when making veracity judgments, but frequently report focusing on the relationship between verbal and non-verbal communication and what was said and how it was communicated. Thus, while verbal behaviour appeared important, autistic observers seemed more sensitive to cross-channel consistency/inconsistency, rather than narrative quality and verbal consistency as indicators of deception.

This distinction may suggest differences not simply in the cues attended to, but in how available information is integrated when making veracity judgments, albeit *post hoc* self-reports are not necessarily a robust indicator of cognitive processes involved in the resultant behaviours. However, this aligns with the qualitative findings, in which autistic participants frequently described perceived discrepancies between facial expressions, body language, and spoken content, even though the quantitative cue-use ratings suggested a greater reliance on verbal information. One possible explanation is that participants attended to multiple sources of information but assigned different weights to these cues. This interpretation is consistent with emerging evidence that reasoning and decision-making in autism may be better characterised by context-dependent differences in information processing than by a simple distinction between intuitive and deliberative reasoning (e.g., Bastan et al., 2025). Rather than suggesting that autistic adults rely uniformly on more deliberative processing, the present findings may instead reflect differences in how available verbal and behavioural information are weighted and integrated during real-time social decision-making under uncertainty. The effectiveness of this approach is therefore likely to depend not only on the strategy adopted, but also on the diagnostic value of the cues being integrated.

We had suggested that autistic adults might approach veracity judgments more systematically or analytically, potentially increasing attention to diagnostic verbal information. However, systematic processing does not necessarily imply reliance on more diagnostic cues. Rather, the consistent application of explicit decision rules to whichever cues are perceived as informative. Here, autistic participants frequently described relying on personal frameworks, searching for discrepancies between communication channels, and evaluating the consistency between verbal and behavioural information. If these rules incorporate behavioural cues or perceived inconsistencies that are themselves weak indicators of deception, systematic application of such strategies may have contributed to the greater truth bias and lower overall decision accuracy observed in the autistic group, albeit this is speculative.

From an applied perspective, our findings may have important implications. Many deception detection approaches encourage observers to evaluate verbal and behavioural information simultaneously. If autistic observers are particularly sensitive to perceived cross-channel inconsistencies, encouraging simultaneous evaluation of verbal and behavioural information may inadvertently increase reliance on behavioural cues, many of which have little diagnostic value for distinguishing truth from deception (see Vrij et al., 2019). Future research should investigate how different sources of information are integrated and weighted during real-time credibility assessment.

Our findings raise the possibility of a more liberal truth response criterion among autistic observers. Truth-default or truth bias is ubiquitous and can aid accuracy when most statements are truthful but increases the likelihood of making an incorrect ‘truth’ decision when deception is present. Retrospective responses to questions regarding types of behaviours attended to may shed some light on these findings, albeit we cannot know if they were causal drivers since responses reflect perceived decision strategies, only. Most autistic observers (70%) described a classic ‘rule’ or a ‘personal framework’ when making a veracity judgment, listing common beliefs or myths about behaviours and deception, and linking specific behaviours to credibility. Autistic individuals are often taught explicit ‘rules’ in childhood to assist in understanding social interactions (Bottema-Beutel et al., 2018). Thus, rule-based social-skills learning may contribute to these findings, albeit this is speculative. Behaviours commonly taught or assumed to indicate discomfort (avoiding eye contact, fidgeting, crossed arms etc.) and/or comfort (smiling, nodding, attentive listening) in others may have been over-generalised as veracity cues. This, may partly account for the different overall confidence levels observed across the two groups. While autistic observers reported higher overall confidence than non-autistic participants, confidence did not vary as a function of veracity condition.

Confidence is not typically a useful indicator of accuracy in applied decision-making, since accuracy and confidence are not necessarily correlated (Dando et al., 2023; Markowitz, 2024; Sandham et al., 2022; Volz et al., 2023). While the present findings do not directly address the relationship between confidence and accuracy, one possible explanation is that confidence may reflect differences in how individuals approach decision-making under conditions of uncertainty, including potentially processing heuristically when cognitive demands are high (e.g., Evans and Stanovich, 2013; Bago and De Neys, 2017; Street and Masip, 2015; Markowitz, 2024). This possibility may be particularly relevant for autistic adults, given suggestions that tasks involving uncertainty can impose increased cognitive demands (Demetriou et al., 2018; Lawson et al., 2017; Tei et al., 2022; Zhou et al., 2024). However, while these interpretations remain speculative and warrant direct investigation in future research, although recent research has noted similarly elevated confidence levels in neurodivergent adult fraud victims (National Trading Standards, 2025).

Discomfort and deception do not necessarily go together since discomfort indicators can be triggered variously and are variously interpreted. Here, lone ‘red flags’ appear important for some autistic observers in assessing credibility. Live, face-to-face judgments are cognitively and socially demanding and under conditions of cognitive loading most lay-people shift from slower, more content-based analysis to faster heuristics. For autistic observers this shift may be stronger. Social and sensory input can sometimes generate higher baseline load and concurrent-task demands (working-memory, cognitive flexibility, switching) potentially making it harder to hold and mentally cross-check details while simultaneously listening to responses to questions (e.g., Melis et al., 2024; Sandham et al., 2022; Dando and Bull, 2011; Dando et al., 2023, 2024). Uncertainty about others’ mental states can encourage reliance on concrete, perceived indicators of discomfort, which may be applied rigidly under stress possibly resulting in a greater pull toward visible, stereotypical cues and a stronger *truth bias.*

Encouraging a structured focus on verbal content, including event-specific detail, relative talking time, and the plausibility of responses, may support more accurate recognition of verbal deception. Explicit, content-focused guidance may encourage a more systematic evaluation of accounts, which could be beneficial for autistic observers. Evidence from non-autistic samples indicates that brief training and cue focused instructions can improve veracity judgments by highlighting the diagnostic value of verbal content relative to unguided observation in some contexts (e.g., Dando and Bull, 2011; Dando and Ormerod, 2020; Sandham et al., 2022; Vernham et al., 2020; Hauch et al., 2016; Driskell, 2012).

Autistic and non-autistic observers similarly rated deceptive accounts as significantly less detailed than truthful accounts, and just over half of autistic observers noted levels of detail, verbal inconsistency and embellishment, stating they were important although very few explicitly mentioned detailedness. Research with neurotypical observers suggests that detailedness can sometimes be a useful heuristic when making relative, within-source comparisons, improving accuracy across modalities (text, transcripts, video, live: e.g., Verschuere et al., 2023) since liars often provide fewer or thinner details than truth tellers in some instances (e.g., Dando et al., 2023, 2024; Palena et al., 2021; Srour and Py, 2023; Vrij et al., 2017). Establishing a comparable baseline for the same speaker improves discrimination in some instances (e.g., Ormerod and Dando, 2015), but in day-to-day situations where like-with-like relative veracity judgments cannot be made, detailedness may have limited diagnostic value, since individual and cultural differences can interfere with between-person effects (Bogaard et al., 2024; Dando et al., 2023; Vrij et al., 2018; Palena et al., 2021; Brennen and Magnussen, 2022). As far as we are aware no research has investigated the detailedness heuristic with autistic participants.

### Limitations and future directions

Accessing hard-to-reach participant groups in adequate numbers necessarily introduces limitations for applied experimental research of this nature. Additional sampling challenges such as victims/survivors of fraud/deception introduces further constraints. Consequently, we did not control for biological sex, our sample comprised adults from the UK and USA general populations, and our adult age range was broad. We also relied on self-reported autism diagnoses, which were not independently verified. That said, to support group characterisation, we included the A-ToM and AQ-10 measures, both of which demonstrated substantial differences between autistic and non-autistic participants, providing convergent support for the distinction between groups. Nevertheless, self-reported diagnoses may not be equivalent to independently verified clinical diagnoses, and the extent to which the present sample is representative of the wider autistic population remains uncertain. Consequently, the applied implications of these findings, particularly regarding potential vulnerability to deception, coercion, and exploitation, should be interpreted with appropriate caution until replicated in samples with independently verified diagnoses.

We did not examine the contribution of A-ToM to deception-related judgments. Thus our observed group differences cannot be fully disentangled from differences in Theory of Mind ability within the current design, although in applied settings, practitioners do not typically have access to an individual ToM abilities and cannot readily assess these characteristics. As such, we have focused on observable group differences in veracity judgments rather than the underlying cognitive mechanisms that may contribute to those differences, as has been reported by others. These constraints constitute a conservative test, but note that reliance on self-reported diagnoses is common in community-based autism research and facilitates the inclusion of autistic adults who may not be engaged with clinical services or research registries.

Pooling sexes obscures differences. Autistic females can present with less overt social difficulties, which may affect how they judge others when making veracity decisions (Bryce and Fraser, 2014; Lim et al., 2022; Parish-Morris et al., 2017). Combining UK and US autistic adult groups may mask culturally driven communication norms, potentially influencing the veracity cues attended to (e.g., Eapen et al., 2023; Kim, 2012; Matson et al., 2011, 2017). Access to social-skills training can also vary within the UK and USA, which may impact veracity judgments (Matson et al., 2012; Sipes et al., 2012). Dichotomising participants as autistic versus non-autistic collapses a heterogeneous spectrum into a binary and obscures within-group variability that may be relevant to veracity judgements.

All interviewees were female, thus the extent to which these findings generalise remains uncertain. Some research suggests gender may influence deceptive behaviour and perceptions of honesty, although evidence is mixed and effects are often small or context-dependent (see Aamodt and Custer, 2006; Elaad, 2022; Capraro, 2018; Gerlach et al., 2019; Lohse and Qari, 2021). Interviewers used an empirically validated questioning technique known to amplify verbal veracity signals including information items provided in response to questions, the proportion of talking time across interviewer and interviewee (Dando and Ormerod, 2020; Dando and Bull, 2011; Ormerod and Dando, 2015; Sandham et al., 2022). These verbal signals were controlled in our stimuli and the verbal differences across veracity conditions do map onto findings from the applied interviewing literature (e.g., Vrij et al., 2017, 2022). Future research avenues might include tighter participant group controls and managing/controlling within group A-ToM variances. Tighter selection and testing of autistic and non-autistic participants may maximise internal validity but would introduce constraints on applied practitioner validity moving forward. Training and transfer random controlled trials with verbal content-focused training, and efforts to replicate the relative judgement paradigm using a both male and female interviewees and a range of verbal interactions would further improve understanding, as would integrating qualitative coding with quantitative decision data.

## Conclusion

Our findings conservatively raise the possibility that some autistic adults might experience challenges in assessing veracity and credibility in certain contexts. However, as we have acknowledged further research is needed since despite constant anecdotal concerns centred on the potential vulnerability of autistic individuals to deception and coercion, very little research has been conducted in this domain (Aller et al., 2024; Griffiths et al., 2019; Maras and Bowler, 2014). Contemporary scams, persuasion attempts, and institutional decision-making contexts are frequently verbal in nature, which may increase vulnerability to exploitation or pressured compliance for some individuals (Federal Trade Commission, 2024; UK Finance, 2024; Ofcom, 2024; National Trading Standards, 2025). Related work has reported broadly comparable patterns in social-evaluative and credibility contexts (Williams et al., 2018; Kurtz et al., 2025; Van Tiel et al., 2021) and recent UK research by National Trading Standards (2025) reports neurodivergent individuals are 50% more likely to be a victim of fraud and that 20% of neurodivergent individuals had been a victim of fraud compared to 13% of neurotypical respondents, underscoring the applied relevance of this line of inquiry moving forward. Structured and explicit guidance regarding the diagnostic value of certain verbal behaviours may be beneficial, since brief, content-focused training has been shown to improve veracity/credibility judgments in non-autistic samples. It seems sensible to expect that similar approaches may also support autistic individuals, although this requires direct empirical evaluation.

## Data Availability

The datasets presented in this study can be found in online repositories. The names of the repository/repositories and accession number(s) can be found at: https://osf.io/utxyc/files/osfstorage?view_only=4b4d93fdeb954adebcc787ccc3efe0f0.
