# Supplementary material for: Autism and detecting verbal truth and lies: veracity decisions, cue selection, judgment confidence, and self-report reasoning
Source: Front Psychol. 2026 Aug 26;17:1871792. doi: 10.3389/fpsyg.2026.1871792 (PMC13553270; doi:10.3389/fpsyg.2026.1871792)
Supplement: Supplementary file 1 [file Table_1.DOCX]

**Supplementary File**

**Post decision open questions**

**B1: *‘****Thinking about the way the person being questioned behaved, describe or list all or any physical/movement behaviours that influenced your truth or lie decision, explaining why these physical/movement behaviours were important to you’*

**B2**: *‘Thinking about the things the person said in response to questions, describe or list all or any verbal behaviours (what people said) that influenced your truth or lie decision, explaining why these verbal behaviours were important to you’*

**Brief Interviewee Participant Paradigm**.

Interviewees were paid £25 to participate and were further incentivised to be deceptive or to be truthful about the events they had all experienced when answering questions, with the offer of an additional payment of £50 if they complied with the relevant instructions.

The interview stimuli comprised two interviews with truthtellers and two with deceivers selected from a larger data set (see Dando & Ormerod, 2020). Each interviewee had been involved in the same live event described below, designed to mimic the experiences of some individuals who might later be interviewed as a witness/person of interest. Interviewee performance data and the paradigm have been reported in full (see Dando & Ormerod, 2020; Dando et al., 2022; Sandham et al., 2023), but for replication purposes and manuscript clarity a brief description of the paradigm is provided.

Participants experienced the target event in groups of six. Embedded in each participant group was a confederate (C1) playing the role of a participant. Confederate 2 (C2) played the role of researcher (C2).

1. Interviewee participants were provided with an information and consent sheet. Following consent, interviewees moved as a group to a seminar room to complete two research questionnaires.
2. While completing the first questionnaire, an unexpected event took place, involving C1 and C2 comprising a verbally aggressive altercation during which a laptop computer was criminally damaged – the entire session, from entering to leaving the event room lasted approximately 25 minutes.
3. Following the event, interviewees were then individually interviewed, and as occurs during investigations in many jurisdictions (e.g., UK, across the EU and some US States, Australia, and Ireland) interviews were digitally audio and video recorded. Interviews followed an interview protocol.

**F-CCE Interview Brief Protocol**

Free Account Phase: This phase is the initial information gathering phase of the interview where interviewees were instructed to explain exactly what happened from the time they entered the classroom until the time they left, in as much detail as possible. The free account was uninterrupted by the interviewer. Once the interviewee had finished speaking, the interviewer moved into the probed questioning phase of the interview.

Probed Question Phase: Interviewees were asked questions regarding i) people involved, ii) what had been said by all involved, iii) the movements of those involved, iv) the environment, and v) finally interviewees were given an opportunity to correct any errors/mistakes and to add any further information. Contact the first author for further information regarding the interview protocol.
